# Automatic Incident Triage in Radiation Oncology Incident Learning System

**DOI:** 10.3390/healthcare8030272

**Published:** 2020-08-14

**Authors:** Khajamoinuddin Syed, William Sleeman, Michael Hagan, Jatinder Palta, Rishabh Kapoor, Preetam Ghosh

**Affiliations:** 1 Department of Computer Science, Virginia Commonwealth University, Richmond, VA 23284, USA; william.sleemaniv@vcuhealth.org (W.S.IV); pghosh@vcu.edu (P.G.); 2 Department of Radiation Oncology, Virginia Commonwealth University, Richmond, VA 23298, USA; michael.hagan@vcuhealth.org (M.H.); jatinder.palta@vcuhealth.org (J.P.); rishabh.kapoor@vcuhealth.org (R.K.); 3 Department of Veteran Affairs, National Radiation Oncology Program, Richmond, VA 23249, USA

**Keywords:** incident learning system, deep learning, automated triage, natural language processing, transfer learning

## Abstract

The Radiotherapy Incident Reporting and Analysis System (RIRAS) receives incident reports from Radiation Oncology facilities across the US Veterans Health Affairs (VHA) enterprise and Virginia Commonwealth University (VCU). In this work, we propose a computational pipeline for analysis of radiation oncology incident reports. Our pipeline uses machine learning (ML) and natural language processing (NLP) based methods to predict the severity of the incidents reported in the RIRAS platform using the textual description of the reported incidents. These incidents in RIRAS are reviewed by a radiation oncology subject matter expert (SME), who initially triages some incidents based on the salient elements in the incident report. To automate the triage process, we used the data from the VHA treatment centers and the VCU radiation oncology department. We used NLP combined with traditional ML algorithms, including support vector machine (SVM) with linear kernel, and compared it against the transfer learning approach with the universal language model fine-tuning (ULMFiT) algorithm. In RIRAS, severities are divided into four categories; A, B, C, and D, with A being the most severe to D being the least. In this work, we built models to predict High (A & B) vs. Low (C & D) severity instead of all the four categories. Models were evaluated with macro-averaged precision, recall, and F1-Score. The Traditional ML machine learning (SVM-linear) approach did well on the VHA dataset with 0.78 F1-Score but performed poorly on the VCU dataset with 0.5 F1-Score. The transfer learning approach did well on both datasets with 0.81 F1-Score on VHA dataset and 0.68 F1-Score on the VCU dataset. Overall, our methods show promise in automating the triage and severity determination process from radiotherapy incident reports.

## 1. Introduction

Radiation therapy (RT) is a popular cancer treatment speciality that involves coordinated interactions between various clinical staff such as, dosimetrists, physicists, radiation therapists, nurses, and physicians. However, misadministration of RT can lead to potentially severe consequences [1,2]. High-risk industries, such as the aviation and nuclear power industries [3], have demonstrated that the incident learning system can prevent such errors. The American Society for Radiation Oncology (ASTRO) and American Association of Physicist in Medicine (AAPM) are professional societies that oversee the accuracy, safety, and quality of RT treatments. In March 2014, these societies started the Radiation Oncology Incident Learning System (RO-ILS) to enable documentation and analyses of incident reports in the radiation oncology domain.

In the wake of RO-ILS, the Veterans Health Administration (VHA) has deployed the Radiotherapy Incident Reporting and Analysis System (RIRAS). The system is being used by the 40 VHA radiation therapy centers as well as the Virginia Commonwealth University (VCU) Health center. RIRAS is a web-based Incident Learning System (ILS) developed by TSG Innovations Inc. and is accessible via the intranet, where any member within the department can submit incident/good catch reports. The taxonomy, data dictionary, and radiotherapy process of care incorporated in the design of RIRAS is based on the AAPM report on “Error Reporting” [4]. Furthermore, RIRAS is fully compliant with the Patient Safety and Quality Improvement Final Rule [5]. RIRAS is built to report all types of workflow events, that includes even minor errors in documentation and processes; such errors may decrease the efficiency of treatments and cause delays besides having other downstream effects.

**Figure 1:**
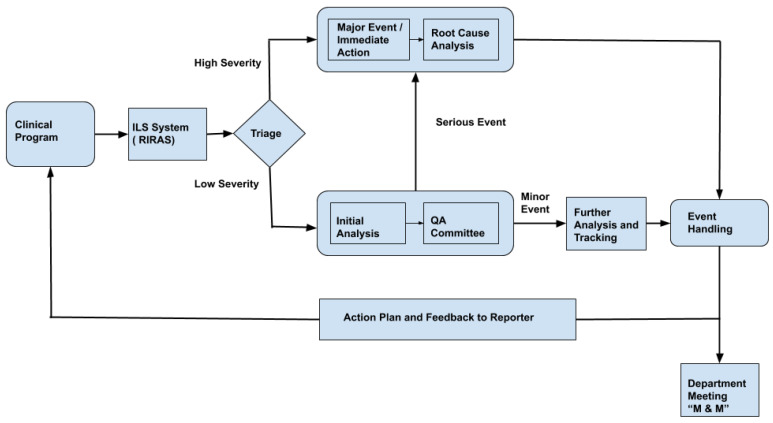
Schematic Representation of Radiation Oncology—Incident Learning System (RIRAS). M & M: Morbidity and Mortality.

Figure 1 shows the typical schematic representation of the RIRAS system. All events reported are reviewed by the ILS committee on a call or face to face interaction; typically such ILS team comprises of medical physicists, dosimetrists, therapists, nurses and physicians. The ILS team completes the analysis form section where event summary titles, error type, causes based on a standard dictionary and safety barriers or quality control measures affecting the event are entered. The event is reported to the chief of the appropriate clinical group if the severity is determined to be high or the ILS team determines that further review is necessary. Otherwise, the ILS committee reviews and codes the events by consensus at their weekly review meeting. Severe incidents require immediate action and root cause analysis (RCA). Understanding the cause of severe incidents helps in preparing an appropriate plan of action. Even the less severe incidents are further analyzed and tracked to avoid similar events. An appropriate action plan and feedback is sent to the incident reporter and professional group so that policy and process can be improved.

Natural language processing (NLP) is a popular technique for analyzing large quantities of clinical texts, notably in medical specialties such as radiation oncology and radiology [6,7]. According to Meystre and Pons [7], the five major categories of application of NLP in radiation oncology are (1) diagnostic surveillance, (2) cohort building for epidemiological studies, (3) query-based case retrieval, (4) quality assessment of radiologic practice, and (5) clinical support services. In this paper, we introduce a sixth category for the application of NLP in radiation oncology: analysis of radiotherapy incident reports. Specifically, we present the use of NLP to automate the prediction of severity from the incident description. As shown in Figure 1, the bottleneck step in the RIRAS system is triaging. We propose a machine learning method to automate the triage process which can thereby reduce the manual efforts needed by the SME to determine the severity; providing an initial prediction of low and high severity with confidence also helps to augment the incident analysis process.

In this work we focused on the safety aspects of radiation oncology. We specifically looked at the triage process in incident learning system. Specific contributions of this work are as follows:

1. We present an approach to automatically identify the severity of the radiation oncology incidents using the textual incident description.
2. We demonstrate that identifying the severity is a challenging problem when it comes to classifying the incidents into the four possible categories using just the incident description. However, merging severity types into two categories (High and Low severities) results in much better classification results considering the incident report data from multiple VHA radiation oncology centers as well as the VCU medical center datasets.
3. We next demonstrated that transfer learning does help in the severity prediction process specifically considering multi-institution data that may each follow a different protocol for recording the incident reports.
4. We show that incident reports are correlated with institutional practices and there is a need for standardized incident reporting guidelines to reduce the subjective incident analysis practices.

The rest of the paper is structured as follows. In Section 3, we present the methods used and details of the data set. Section 4 describes the results and in Section 5, we present the discussion and conclusion. In the final section, we present the limitations in our approach that can motivate future work.

## 2. Background

Healthcare incident reports, including the radiotherapy incidents submitted into the RIRAS software, are similar to the safety reports of various industrial environments in that their narratives are reported in an unstructured free-text format. Free text, while convenient for the reporter, presents a challenge for data aggregation and requires suitably-qualified personnel to read and analyze. However, due to the lack of dedicated incident-analysis personnel, minor incident reports in healthcare often accumulate, as resources are used to deal with front-line issues that are typically considered more urgent.

To the best of our knowledge, there is no work reported in the field of radiotherapy to identify the severity of the incidents reported using incident description. However there have been well reported research in other industries such as aviation, and nuclear [8,9,10,11,12] to classify the incidents reported in the respective fields. In healthcare there has been successful work done in classifying the verbal autopsies [13]. A team in Canada has done a study on identifying the incident types from Canadian medication incident report [14]. Another team in Australia performed more extensive study predicting the two types of patient safety incidents: incorrect patient identification and inadequate clinical handover [15]. Hence, there is an urgent need for creating an actionable learning-based incident reporting system in healthcare [16].

## 3. Methods and Materials

### 3.1. Incident Severity Types

The AAPM (professional society of Medical Physicist in the US) formed a working group on Prevention of Errors in Radiation Oncology where a panel of experts developed consensus recommendations considering five key areas: data elements, definitions, severity scales, process maps, and causality taxonomy [4]. RIRAS was implemented following these recommendations. Following are the important terminologies related to ILS:

- **Incident:** refers to events that are unintended or unexpected in the realm of standard clinical operations. Such events may cause adverse effects on equipment, healthcare providers or patients.
- **Near Miss or Good Catch:** refers to unplanned events that could potentially cause a damage, illness or injury, but did not actually do so. However, such near misses were only averted due to good fortune. Such events are mostly labeled by “human error”, while faulty systems or processes may exaggerate the harm, and needs to be studied better. Other terms used for such are “close call”, and for moving objects, “near collision”.
- **Unsafe Condition:** refers to hazardous work environments, circumstances or physical conditions that may potentially lead to different incidents.

In the VHA, the National Radiation Oncology Program (NROP) consists of 40 facilities treating over 12,000 patients annually within the system, and an additional 14,000 outside of the system. As the rate of errors has been estimated to occur as frequently as 1 per 600 patients [17], the utilization of ILS can provide a means of gathering and analyzing incident data so that patient safety and workflow process improvements can be implemented and the effects of such changes tracked over time. For multi-institutional programs such as the NROP, aggregating incident reports from all facilities into a single database increases the effectiveness of incident learning and allows for the assessment of systematic errors and trends as well as national standardization of policies and procedures. Based on the recommendation of AAPM, NROP defined the reasoning behind the severity categorization and explained what constitutes of low to high severity. Reports were subsequently categorized based on four levels of severity: A through D. Explanations for these incident severity categories are shown below:

- **Level A**: It is a significant event or near miss with a potential for a medical event or serious patient injury, as well as a repeat of a Level B event. The problem has an urgent need for correction and may impact multiple patients or Radiation Oncology processes. Level A incidents require a full Root Cause Analysis. The Lead Responder for a level A incident will typically be a medical physicist. Very few (<2%) incidents should fall into this category.
  Example: A patient is treated at the wrong site. The Lead Responder would be a medical physicist appointed by the Director of Clinical Physics.
- **Level B:** It is a significant event or near miss that did or could result in a dose deviation >5%, a significantly larger than intended dose outside the treatment field, a treatment delay of greater than one day, or a similar scenario that is neither a Medical Event nor poses a risk of serious patient/staff injury. The problem should be confined to a single process step and could likely be promptly addressed with an Apparent Cause Analysis. The Lead Responder for a level B incident will either be a medical physicist or a department lead. Few (<5%) incidents will fall into this category.
  Example: A case is planned and treated for five fractions (out of 20) with an improperly expanded contour that is 5 mm larger than intended by the physician. The Lead Responder would be the Director of Dosimetry.
- **Level C**: A minor incident, near miss, or condition that warrants an appropriate response from a department lead, who is typically the Lead Responder. The level of the response will be up to the department lead, but the response must be reported back to the Quality Assurance (QA) committee. Many incidents will fall into this category.
  Example: A case is planned and prepared for treatment assuming 5 mm bolus. The physician opts not to use the bolus, and only the monitor units are not recalculated before treatment approval. The Lead Responder could be the Director of Clinical Physics.
- **Level D**: A very minor incident, near miss, or condition that warrants awareness by the department lead. The level of the response will be up to the department lead, and there is no mandate for them to report back to the QA committee. The incident will be logged within RIRAS for trend tracking purposes.
  Example: A field is mislabeled in a plan. The Director of Dosimetry is informed.

### 3.2. Dataset

RIRAS is a web-based ILS deployed on the VHA radiation oncology centers intranet and VCU intranet in early 2014. It was designed to collect good catch data and adverse events, besides analyzing their causes and contributing factors, and finally, to prevent possible occurrences in the future. This system provided a platform to report the adverse events across 40 VHA radiotherapy treatment centers. We collected data from both sources, which consisted of incidents that were triaged into four levels of severity, namely, A through D, where A is most severe, and D is least. From here on, the dataset collected from VHA centers and VCU radiotherapy center will be referred to as VHA data and VCU data, respectively. Table 1 shows the sample examples of incident descriptions reported and their respective severities assigned.

**Figure 2:**
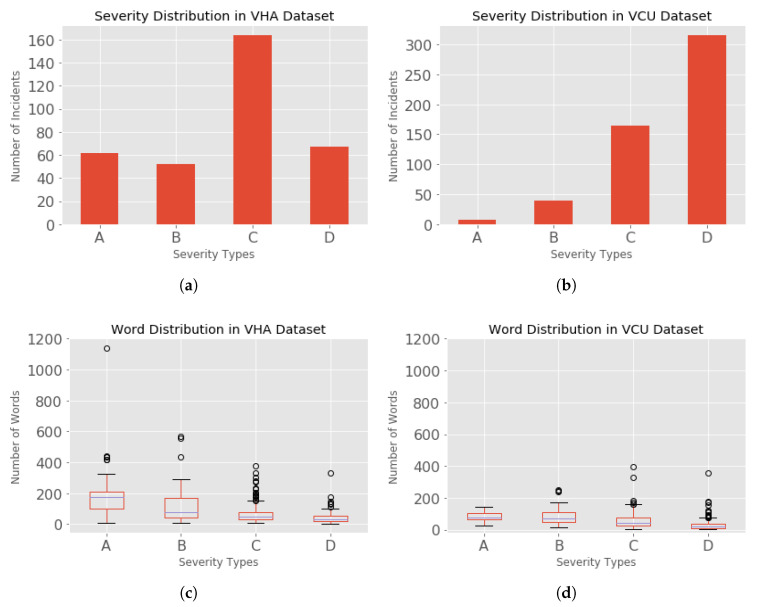
Dataset Distributions: (**a**) Severity Distribution in VHA dataset. (**b**) Severity Distribution in VCU dataset. (**c**) Word Distribution in VHA dataset. (**d**) Word Distribution in VCU dataset.

**VHA Data set:** The VHA clinical reporters entered incidents in the RIRAS since 2014. For the time period between 2005 and 2014, the incident reports were collected for only high severity (level: A) incidents. These reports were collected by mostly emailing the VHA’s National Health Physics Program office who logged the reports in excel spreadsheets. These reports (46 reports) were entered into RIRAS in 2015. For the purposes of this analysis we used the data collected till 2017. A total of 530 incidents were reported across the VHA centers at the time when this data was collected, in which 345 incidents were analyzed by the subject matter experts and the incident analysis reports were assigned severities. The incidents distributed based on the severity in VHA dataset is as shown in Figure 2a, where the incidents are distributed as A (62), B (52), C (162), and D (67). A total of 185 incidents were not analyzed and hence were missing the severities; such non-analyzed incidents cannot be used in our classification task.

**VCU Data set:** The incidents collected at VCU were between 2014 to 2019. A total of 540 incidents were reported, among which 7 were not analyzed by the subject matter experts. The incidents were distributed based on their severity as shown in Figure 2d, where the incidents were distributed as A (9), B (40), C (165), and D (318). A total of 7 incidents were missing severities.

### 3.3. Model Selection

In this section, we describe the model selection techniques using traditional machine learning and deep learning approaches with model fine tuning and transfer learning.

### 3.4. Traditional Machine Learning

We first pre-processed the textual data from the incident reports. The next step was to select the appropriate machine learning algorithm for which we tested different types of algorithms to predict the severity of the incidents. We next identified the features from the text to build the corresponding feature vectors which are necessary for any supervised machine learning algorithm.

**Figure 3:**
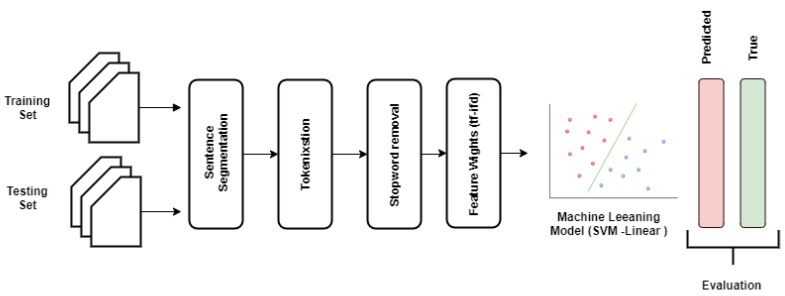
Pictorial representation of the traditional machine learning severity classification pipeline. SVM-Linear: Support vector machine learning with linear kernel, *tf-idf*: term frequency—inverse document frequency. Blue and red dots indicates classes.

Since machine learning algorithms require numerical data, we next converted the textual data into numerical features. This involves the following major steps [18]: (1) tokenization, (2) feature set generation, and (3) vectorizing the features with different feature weight calculation techniques. To this end, we applied the following steps in developing the proposed traditional machine learning pipeline (as shown in Figure 3).

#### 3.4.1. Data Splits

One of the important steps in building a machine learning model is to evaluate it properly. If the model is evaluated on the same data on which it is trained, there is a chance that it may perform well on the training data but poorly on future data. Therefore, it is recommended to build a model by splitting the data into three sets, the training set, validation set and test set. Using the separate data for evaluation not seen during training lets us test if the trained model is not over trained. Once the final model is prepared, the test data set is used to test the model with unseen data (not seen during training and not used as validation).

#### 3.4.2. Data Preprocessing

All incident descriptions were first processed using NLTK (python library for text processing) [19]. The following procedures were applied:

- ***Data Cleaning:*** Removing the unnecessary parts of text. In our dataset, we removed the characters “&amp;quot”, “&amp;&amp;”, which were added to the text when collecting the data from XML files.
- ***Tokenization:*** It is the process of splitting the long string of text (sentences) into tokens (words). These tokens are used as features. We used NGram tokenization to produce unigram, bigrams, and trigrams [20]. Unigrams are also known as bag-of-words representing individual terms that occur in a document (e.g., “surgery”, “prostate”, “dosimetry”), bigrams and trigrams represent the consecutively occurring two or three terms in a document (e.g., patient scheduled, patient re-scanned, patient planned radiation therapy), which help capture the semantics of text; one such example is negation (e.g., no pain).
- ***Text Normalization:*** It is the process of converting terms occurring in text into one form. We used lower case normalization to ensure that all the words occurring in different forms are represented as one (e.g., Patient, PATIENT, patient, and pAtient are converted to “patient”) [21].
- ***Stopword Removal:*** It is the process of identifying and removing more frequently occurring words from the text. We considered removing commonly occurring English language words (e.g., a, the, it, what, why, she, etc.), which hold no classification value [20]. We used general English language stop words provided in the NLTK Package. This technique is commonly used in information retrieval and NLP document classification implementations [21].
- ***Term frequency filtering***: It is the process of identifying the infrequently appearing words in the corpus [22], which helps with reducing the feature vector size. We have used a minimum term frequency of 5 as cutoff.
- ***Feature Weighting Techniques***: We used three types of feature weighting methods as shown below:
  1. ***Term Presence (tp):*** The term weight assigned to 1 or 0 based on the presence or absence of a term in the given text.
  2. ***Term Frequency (tf):*** The weight of the term calculated based on the number of times a term occurs in text over the total number of terms in that text.
  3. ***Term Frequency-Inverse Document Frequency (tf-idf):*** It is calculated by multiplying the two components *tf* and *idf*. It reflects the importance of a term in a text within a collection of documents [23].
    (1) $${tf\text{-}idf}_{t,d}=\left(1+\log{tf}_{t,d}\right)\cdot \log\frac{N}{{df}_{t}}$$
    Equation (1) shows the mathematical formulation of *tf-idf*. Here *t* is the term, *d* is the document, *tf* denotes the term frequency, *df* is number of documents, ${df}_{t}$ denotes the number of documents in which the term, *t*, appears, and *N* is the total number of documents.
- ***Vectorization***—It involves using the above steps to extract features and weights to generate uniform vector representations of each report. Each feature weighting technique (shown above) was used to create three types of feature vectors. One such feature weighting technique is *tf-idf*; *tf-idf* assigns the weight to the term based on its frequency in a document, and its appearance in all documents in the corpus. The assigned weight indicates the relevancy of that term to the document when classifying the documents into different classes [21,24,25]. The higher value of the term indicates its higher importance. The term frequencies are normalized so that longer documents do not skew the results [26].

**Example:** Consider the below incident reports; we have used a short description for explanation purposes. In real-world datasets, the incident descriptions are longer.

1. One of the treatment field was miss labeled
2. Plan not sent to RadCalc
3. Esophagus structure was not interpolated
4. Wrong plan was sent to RadCalc

From the above documents, after removing the English language stop words, the following uni-gram features are extracted.

Features: [esophagus, field, interpolated, labeled, miss, plan, radcalc, sent, structure, treatment, wrong].

Feature vectors for above samples (using the *tf-idf* weighting method).

$$\begin{array}{cccccccccccc} features= & [esophagus & field & interpolated & labeled & miss & plan & radcalc & sent & structure & treatment & wrong]\\ 1= & [0 & 0 & 0.5 & 0 & 0.5 & 0.5 & 0 & 0 & 0 & 0.58 & 0]\\ 2= & [0 & 0 & 0 & 0 & 0 & 0 & 0.58 & 0.58 & 0 & 0 & 0]\\ 3= & [0.58 & 0 & 0.58 & 0 & 0 & 0 & 0 & 0 & 0.58 & 0 & 0]\\ 4= & [0 & 0 & 0 & 0 & 0 & 0.47 & 0.47 & 0.47 & 0 & 0 & 0.59] \end{array}$$

#### 3.4.3. Classification Algorithms

Next, we explain the classification algorithms that are tested to select the best algorithm for the traditional machine learning pipeline.

- ***k-Nearest Neighbors (kNN):**k*NN is a simple classification algorithm which involves finding the *k* nearest neighbors in the dataset [27]. Nearest neighbors are determined using the distance metrics such as Euclidean or Manhattan distance. *k* is the only parameter that needs to be set in the *k*NN algorithm. Recommended *k* value is $\sqrt{n}$, where *n* is the number of data points. However, other *k* values may depend on the properties of the dataset [28].
- ***Logistic Regression (LR):*** Logistic regression is a simple linear algorithm that takes in a vector and converts it to the probability ranging between 0 and 1. It uses the sigmoid function to convert the value. For binary classification, a cutoff value is used to decide the class label. It is easy to interpret due to its linear nature. Even though it is predominantly used for binary classification, it can also be used for multi-class classification.
- ***Support Vector Machines (SVM):*** Support vector machines make use of a hyperplane or set of hyperplanes to distinctively classify the data points. Linear SVM makes use of maximum-margin hyperplanes to classify the linearly separable datapoints [29]. Alternatively, non-linear SVM uses the function to map the input vector to a high-dimension or infinite-dimension vector space and determines the hyperplane in the new space to classify the data points [30]. It has been previously observed that SVMs have consistently outperformed many other classifiers in text categorization problems, and they are less prone to the imbalanced data sets [31].
- ***Random Forests (RF)***: Random Forests consists of multiple decision trees, but each tree can only be split based on the randomly selected subset of features from the randomly selected samples. For each tree, different subset of samples and subset of features are selected randomly. For classification, majority voted label is considered as the predicted label [32].

#### 3.4.4. Evaluation Metrics

To evaluate our model we considered macro-averaged precision, recall, and F1-Score. A macro-averaged metric of a model will compute the metric independently for each class and then take the average, whereas a micro-average will aggregate the contributions of all classes to compute the average metric. Macro-averaged Precision, Recall, and F1-Score can better capture how well a classifier can identify cases that it does not see often, which is highly important in real-world settings. Mathematical expressions of each of these metrics are shown below.

(2) $$Precision=\frac{TP}{TP+FP}$$

(3) $$Recall=\frac{TP}{TP+FN}$$

(4) $$F1-Measure=2\cdot \frac{Precision\cdot Recall}{Precision+Recall}$$

Here, *TP* is true positive, *TN* is true negative, *FP* is false positve, and *FN* is false negative counts.

Results are also presented using a confusion matrix which shows the number of correct and incorrect predictions as summarized with prediction counts between each class. It provides insight not only into the errors being made by the classifier but more importantly, the types of errors that are being made. All the metrics mentioned are computed from values in the confusion matrix.

#### 3.4.5. Initial Model Selection

The extracted incident reports were used to train machine learning classifiers with Python’s scikit-learn (version 0.21.3) [33]. The labeled incident report corpus was stratified as 80:20 as training and test split. A total of 276 (80%) incident reports were used for model training and 69 (20%) for model testing to characterize the model performance.

In our initial work, to test the viability of predicting all four severities, we built four different models by combining severities as below [34]:

- Model-1: We considered incidents with severities A and C.
- Model-2: We combined A&B as high and C&D as low severities.
- Model-3: We considered only B and D severity.
- Model-4: All 4 severities, A, B, C, and D are considered as separate.

These models provide insight into our methods’ ability to find patterns when incidents with different severities are considered. We built above mentioned four models with SVM-linear classification algorithms, and NGram features with *tf-idf* feature weights. Table 2 shows the results of these four models. We observed that Model-1 and Model-3 achieved an F1-Score of 0.87 and 0.78 respectively; we can infer that incidents A & C (Model-1) and B & D (Model-3) have better patterns to classify incidents. The poor performance of Model-4 indicates that there is a lot of similarities between the A & B and C & D severities in the confusion matrix. Model-2 achieves the F1-Score of 0.81. It is clear from the results that predicting all the four categories is difficult based on our current datasets. However, categorizing incidents into high (A & B) and low (C & D) severity (Model-2) is viable.

Hence, we used Model-2 for building the automated triage system. To select the best classification algorithm to build the final model, we applied the above explained steps to build the severity prediction model. Figure 3 shows the pictorial representation of the classification pipeline; for a general review of natural language processing approaches applied in a clinical context, refer to [35]. Five different classification algorithms were used: *k*-Nearest Neighbors (*k*NN) [27], SVM-Linear [29], SVM-RBF [30], Random Forests [32], and Logistic Regression [36] with feature extraction and weighting methods. Standard macro-average, Precision, Recall, and F1-Score are used as evaluation metrics for discrimination on the training and test sets. Table 3 shows the results of the initial model selection. We observed that SVM with linear kernel consistently performed well with all feature vector generation methods. In all combinations of algorithms and features, SVM with linear kernel algorithm and *tf-idf* features performed the best with an F1-Score of 0.808. With this observation, we chose the *tf-idf* and SVM-linear to build our final model.

### 3.5. Traditional Machine Learning vs. Transfer Learning

Traditional machine learning refers to training a model on a particular task (say, text classification) from one domain and expecting it to perform well on unseen data from the same domain. Whereas, transfer learning refers to the use of a model that has been trained to solve one task (e.g., language modeling: predict next word in a sentence) as the basis to solve some other or somewhat similar problem (text classification) Pan and Yang [37]. It also refers to the training a model with a large-scale dataset and next using this pre-trained model for the same task with different dataset and labels. The computer vision domain popularized transfer learning with the ImageNet dataset.

**Figure 4:**
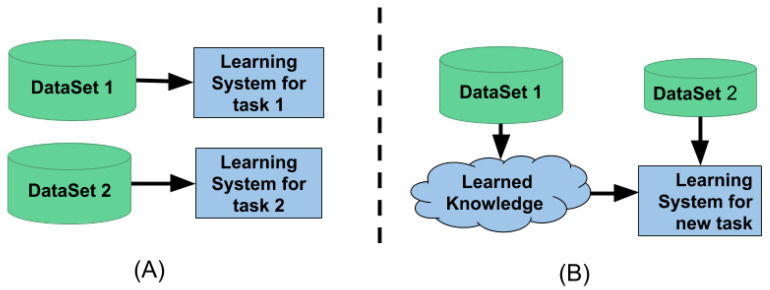
(**A**) Traditional machine learning system (**B**) Transfer Learning system.

Figure 4A shows the traditional machine learning setup. This method is isolated and performs single-task learning. It is not possible to use the knowledge from one task to learn the new task. Traditional machine learning also needs a lot of data to learn the given task. Whereas Figure 4B shows the transfer learning setup. This setup utilizes the knowledge learned from one task to learn a new task; because of the knowledge transfer, it requires less data and computation time to learn a new task.

#### Transfer Learning

A simple and extremely popular transfer learning technique in NLP is to use the word2vec embeddings, which uses a single layer of weights from the trained model. However, full neural networks in practice contain many layers, and using transfer learning for a single layer is clearly only scratching the surface of what is possible. From the immediate past, one such technique that fine tunes the full network for transfer learning on textual data is the universal language model fine tuning (ULMFiT) [38].

### 3.6. Universal Language Modeling and Fine Tuning

**Figure 5:**
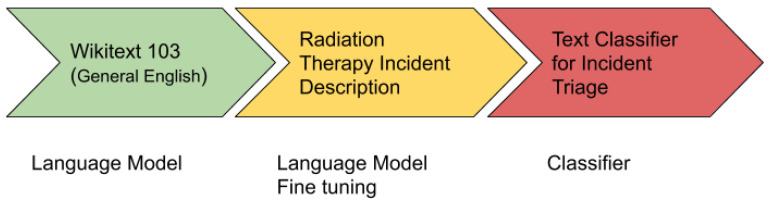
High level Universal Language Model Fine-tuning (ULMFiT) approach used for incident triage.

The ULMFiT is one of the revolutionary algorithms in the field of NLP for knowledge transfer used for text classification. It uses all the layers of a neural network for transfer learning. Figure 5 shows the architecture of ULMFiT.

The ULMFiT has three main steps:

1. General Domain Language Modeling: In the first step, an unsupervised language model is trained on a large corpus to generate a general-domain language model. For this, a pre-trained general-domain English language model was used [38], which is trained with state of the art language model AWD LSTM on Wikitext-103 [39].
2. Target Task Language Model Fine Tuning: In the second step, the general domain language model is fine-tuned with the domain/target specific dataset. A pre-trained general-domain language model allows the target task language model to converge faster and results in a robust language model even for small target datasets. A pre-training provides a robust representation for uncommon words in the target training dataset.
3. Target Classifier Fine Tuning: In the third and final step, it adds two additional linear blocks to the pre-trained language model. The first linear layer takes the pooled last layer of the language model as input on which it applies ReLU activation. The last layer is a fully connected layer having softmax activation that provides the target classes’ prediction probability.

## 4. Results

In this research, our goal was to augment the triage process in RIRAS by predicting the severity of the incident using the textual description of the incidents reported. We used two different approaches to predict the severity of the reported incidents: a traditional ML and transfer learning approach with the more advanced algorithm called ULMFiT. Below we describe the results from each of these approaches.

### 4.1. Traditional ML Results

**Figure 6:**
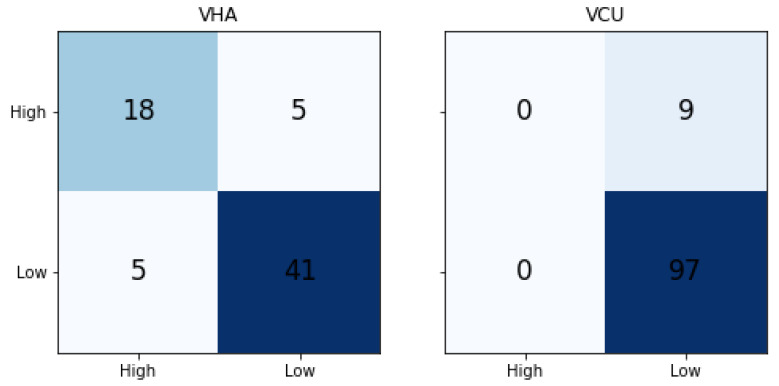
Traditional ML Results Confusion Matrix. Left confusion matrix is for VHA test set and right is for VCU test set. Diagonal indicates the correctly predicted class count.

From the initial model selection results, we observed that SVM-linear performed best in comparison with others. Hence, we used the SVM-linear to build the final model. We built separate models for VHA and VCU datasets. Table 4 shows the traditional ML results. We compared the results with the majority label baseline (MLB Baseline) model. In the MLB baseline, all the predictions are done as a label that occurs the majority of the time. The metrics are calculated based on the majority label. In a balanced binary classification model, the random probability of predicting a correct class is 50%, but both the datasets used in this work are imbalanced. Hence, we compared the results with the Random and MLB baseline. The VHA dataset model achieved 0.80, 0.77, and 0.78 of precision, recall, and F1-Score, respectively. When compared to the MLB baseline, it achieved much better results. Whereas for VCU, we noticed that SVM-Linear results are the same as the MLB baseline, indicating that the model was not able to learn the classification patterns from the training data. Figure 6 shows the confusion matrix of traditional ML results for both VHA and VCU. We noticed that for the VCU dataset, the ML model assigned the Low severity (majority label in the training set) to all test set instances.

### 4.2. Transfer Learning Results

Table 5 shows the results for different models built with ULMFiT. As explained in Section “Transfer Learning”, transfer learning is a way to utilize the knowledge learned from one task into another task. In this research, we used ULMFiT to build the transfer learning based approach to predict the severity of incident reports in radiation oncology. ULMFiT involves building the language model (LM) and use it in the classification model.

In order to test the effects of data source on the models’ ability to predict the severity of the incident reported using the descriptions, we built three different LM models based on the data source: VHA, VCU, VHA_VCU. Next, we trained the separate classification models with VHA and VCU datasets by taking knowledge from the LM models. This provided us with (3 X LM model) X (2 X Classifiers) = 6 pipelines to test for each data source, and a total of 12 models for VHA and VCU. Table 5 shows the transfer learning results. The results reported are macro-averaged precision, recall, and F1-Score.

**Figure 7:**
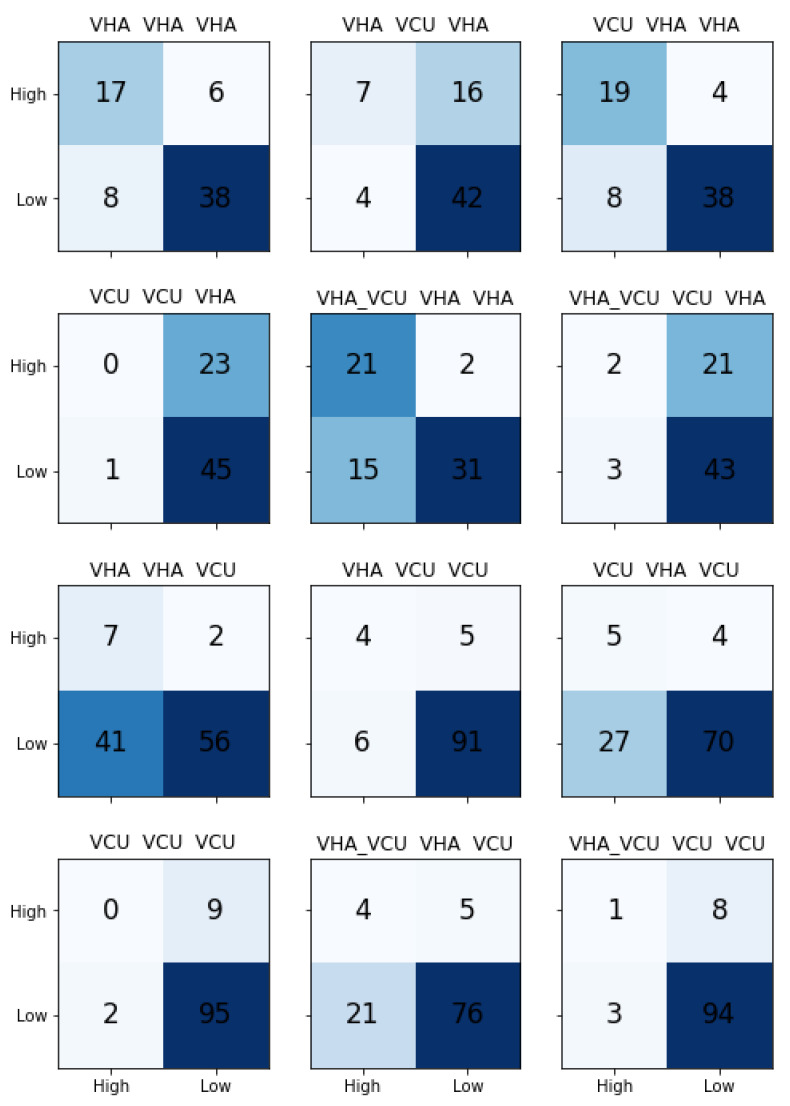
Transfer Learning Results: Confusion Matrix for each model in test dataset. Title in each confusion matrix indicates the respective model. Top two rows (six models) is for VHA testset and bottom two rows (six models) for VCU testset. Diagonal indicates the correctly predicted class count.

We observed that transfer learning results are comparably better than traditional ML learning results. For the VHA test set, we noticed that the pipeline with VCU LM model and classification model trained with VHA achieved the best results. LM models trained separately with VHA, VCU, and VHA_VCU performed similarly for the VHA test set. It is clear from the results that the classification model needs to be trained with VHA data to predict the VHA test set. Transfer learning models performed well for the VCU dataset with precision 0.67, recall 0.69, and F1-Score of 0.68 compared to the traditional ML model. Figure 7 shows the confusion matrices for all the models. The model with LM trained on VHA data and classifier trained on VCU data performed better on the VCU test set.

## 5. Discussion

In this paper, we presented an approach to predict the severity of the radiation oncology incidents. The purpose of this work is not to replace the manual triage process, but rather, augment it by predicting the severity of the incident with reported description and provide the recommendation to the subject matter experts on the likelihood of an incident being of low or high severity. To do that, we used NLP techniques and ML algorithms to build the automated triage pipeline. We used traditional ML and transfer learning approaches. The datasets used in this work come from two different sources; they are similar, yet have different characteristics. We noticed that the distribution of incidents based on the severity type is different in VHA and VCU datasets; there are fewer High severity incidents in the VCU dataset compared to the VHA dataset even though the total number of incidents in VCU are higher than VHA. We noticed that the descriptions of the incidents reported in the VHA dataset are longer on average compared to the incident descriptions reported in the VCU dataset. The length of the incidents also correlates with the severity of the incidents. The High severity (A & B) incidents, on average, have long descriptions compared to the Low severity (C & D) incidents. It does not mean that the length of the description of the incident indicates the severity of the incidents. However, we believe it may be because the incident reporters tend to describe incidents in detail if they deem the incident is severe. The difference in length of descriptions may be due to the institution type and practice at those institutes. VHA incidents are coming from 40 VHA treatment centers, whereas VCU is a single institute. NLP makes use of the words in the description to find the patterns of the specific severity. Hence, a well-explained description is always better than a short one. Talking to SMEs, we have learned that some times just incident description provided is not enough to infer the severity; they always reach out to incident reporters for more information before analyzing the incident and assign severity. Thus, we believe that there is a need and opportunity to build guidelines on reporting practices. All the staff who use the RIRAS system to report incidents needs to be aware of guidelines and follow the instructions while reporting an incident.

### 5.1. Strengths and Limitation

This study makes the following contributions. First, it is the first study of its kind to use radiotherapy incident reports (RIRAS) to build the automated incident severity determination pipeline using ML/NLP. Second, it compared the use of traditional and transfer learning in the context of incident triage.

This work has many limitations. The datasets used are comparatively small with only 800 reports from two sources (VHA and VCU). There is also a high class imbalance in our datasets, which makes it difficult to train an accurate model because the model sees more samples for one class and fewer samples for other classes. Hence, acquiring more balanced data is needed. Furthermore, it remains unclear if there were substantial changes between the VCU and VHA datasets. Model performance is compared with gold standard, which is a manually annotated dataset. Hence, the model performance evaluation depends on the quality of these manual annotations. Multiple annotators manually annotating the same incidents reports and measuring the inter-annotator agreement might improve the manual annotations. However, in our datasets, each incident was annotated by a panel of SMEs and there is only a single annotation for each incident. Even though there are instructions to analyze the incidents, the triage process is very subjective, which makes it difficult for algorithms to capture the subjectivity with single annotation. Models were trained and tested using only incident reports, where the incident severities were assigned based on the information gathered as follow-up with the incident reporter. It was also noticed by the SME panel that sometimes the incident descriptions did not provide a complete picture, which makes it challenging to understand the appropriate severity solely based on the report.

### 5.2. Comparison with Previous Work

While ML and NLP based methods have been widely used to analyze incident reports from other domains, such as aviation [40], they have only been scarcely used in the healthcare domain before [15]. Straightforward comparison of our work with others is not possible because of the following two reasons. First, there has been no prior work related to the radiation oncology incident severity prediction using ML and NLP. Second, related work in healthcare incident analysis is more focused on other types of incident reports, where such incidents were recorded as free text. For example, Wong and Akiyama [14] analyzed 227 medication incident reports using a logistic regression based classifier to categorize the incident types based on adverse drug effects. Similarly, Wang et al. [41] used an integrated ML and NLP based pipeline to categorize incident reports related to patient safety; however, their method performed poorly in properly classifying the severity levels. Finally, another related work in the healthcare domain considered verbal autopsies for text-based classification [13] with good accuracy; such autopsies bear some resemblance to incidence reports. However, none of these works are directly comparable to our proposed method which considers incident reports from the radiation oncology domain for automatic classification of severity levels and hence precludes any direct comparison with prior work.

### 5.3. Future Work

We have used ULMFiT in our current work for the transfer learning method. In the future, we would like to compare this method with similar approaches such as ELMo [42], OpenAI GPT [43], and BERT [44]. For the BERT based model, we will use the publicly available pre-trained model on clinical BERT as our base [45]. Results will be compared and the best performing model, which will be used to implement the final automated triage pipeline.

ULMFiT and BERT base models are trained on huge amounts of general English text. In this work, data was considered from the very niche field of radiation oncology. We will fine tune these models with data from RIRAS to provide the context. This can improve the over-all performance of the model.

Understanding why incidents occur may be more important for effecting change than understanding what incidents have occurred. Further studies exploring the ability of NLP to classify incident reports by contributory factors could offer more learning opportunities.

As shown in the methods, we do not have enough instances for each severity type (for 4 types). Hence, we combined the severities to form new categories representing low and high severities. In the future, we would like to collect data for all types of severities to build the automated triage system that can closely resemble the real triage process.

## 6. Conclusions

Incident reports in the radiation oncology domain provide very useful information to analysts and subject matter experts to decide on the right course of action for incidents. With the current trends in digitization of medical data (such as, incident reports) and automation of operations and logistics (such as our proposed automated incident triage and prioritization module), artificial intelligence related methods have become a necessity. In this paper, we presented a deep learning based ULMFiT model that can effectively identify the incidents based on the initial report and narrative. We demonstrated that this transfer learning based approach outperforms standard supervised machine learning based approaches for classifying narratives. Our work provides encouraging results towards the end goal of a fully automated incident triage and prioritization system in the future. Additional data from the national safety registry RO-ILS should help to improve the accuracy of our proposed model and provide human-level fidelity and performance. Our models can also work on retrospective data on incident reports to automatically classify the incident severity and provide rapid summarization of past events for subsequent data driven research studies in the future.

## Figures and Tables

**Table 1:** Examples of Incident description and respective Severity assigned by Subject Matter Experts.

Incident Description	Severity
The patient on the EMR screen was not the patient called for treatment. During set up the radiation therapist noticed that the patient on the table is not the patient selected on EMR. Introduced new policy of double checking the patient ID by therapists.	High (A or B)
Spinal cord and Brainstem max doses were incorrectly recorded in dose summary spreadsheet and in paper chart and Aria printouts. Aria dose recording paper chart and Aria PDF were corrected.	Low (C or D)

**Table 2:** Initial model selection results from the severity categorization model for different combinations of severities. Results reported are macro-averaged.

Models	Severities	Precision	Recall	F1-Score
Model-1	A and C	0.86	0.87	0.87
Model-2	A & B and C & D	0.83	0.80	0.81
Model-3	B and D	0.80	0.77	0.78
Model-4	A, B, C, and D	0.53	0.56	0.53

**Table 3:** Model-2 selection results for severity categorization using traditional machine learning approach. Results reported are macro-averaged. Bold values indicated best results generated by feature weights and algorithms combination.

Dataset	Features Weights	Algorithm	Precison	Recall	F1-Score
		SVM_RBF	0.809	0.519	0.418
		SVM_Linear	**0.792**	**0.698**	**0.705**
	*tp*	Random_Forest	0.856	0.685	0.686
		Logistic_Regression	0.792	0.698	0.705
		KNeighbors	0.304	0.500	0.378
		SVM_RBF	0.797	0.655	0.649
		SVM_Linear	**0.815**	**0.735**	**0.747**
**VHA**	*tf*	Random_Forest	0.837	0.729	0.740
		Logistic_Regression	0.815	0.735	0.747
		KNeighbors	0.813	0.537	0.454
		SVM_RBF	0.720	0.562	0.512
		SVM_Linear	**0.835**	**0.798**	**0.808**
	*tf-idf*	Random_Forest	0.818	0.692	0.696
		Logistic_Regression	0.759	0.599	0.571
		KNeighbors	0.680	0.664	0.668
		SVM_RBF	0.458	0.500	0.478
		SVM_Linear	0.458	0.500	0.478
	*tp*	Random_Forest	0.458	0.500	0.478
		Logistic_Regression	0.458	0.500	0.478
		KNeighbors	0.458	0.500	0.478
		SVM_RBF	0.458	0.500	0.478
		SVM_Linear	0.460	0.500	0.475
**VCU**	*tf*	Random_Forest	0.458	0.478	0.478
		Logistic_Regression	0.460	0.490	0.473
		KNeighbors	0.458	0.500	0.478
		SVM_RBF	0.458	0.500	0.478
		SVM_Linear	0.460	0.495	0.475
	*tf-idf*	Random_Forest	0.458	0.500	0.478
		Logistic_Regression	0.458	0.500	0.478
		KNeighbors	0.457	0.490	0.473

**Table 4:** Traditional machine learning approach results for Model-2 (Low vs. High severity prediction). Reported results are macro averaged. MLB: majority label baseline.

Models	Data Source	Precision	Recall	F1-Score
Random	−	0.50	0.50	0.50
MLB Baseline	VHA	0.33	0.50	0.40
	VCU	0.458	0.500	0.478
SVM-Linear	VHA	0.80	0.77	0.78
	VCU	0.458	0.500	0.478

**Table 5:** Transfer Learning Results for Model-2. First six rows for VHA test-set models and last six rows are for VCU test-set. Results reported are macro-averaged. Support indicated the total number of samples in test-sets. LM: Language Model. Bold values indicates the best results generated.

LM	Train	Test	Precision	Recall	F1-Score	Support
VHA	VHA	VHA	0.77	0.78	0.78	69
VHA	VCU	VHA	0.68	0.61	0.61	69
VCU	VHA	VHA	**0.80**	**0.83**	**0.81**	69
VCU	VCU	VHA	0.33	0.49	0.39	69
VHA_VCU	VHA	VHA	0.76	0.79	0.75	69
VHA_VCU	VCU	VHA	0.54	0.51	0.46	69
VHA	VHA	VCU	0.56	0.68	0.48	106
VHA	VCU	VCU	**0.67**	**0.69**	**0.68**	106
VCU	VHA	VCU	0.55	0.64	0.53	106
VCU	VCU	VCU	0.46	0.49	0.47	106
VHA_VCU	VHA	VCU	0.55	0.61	0.54	106
VHA_VCU	VCU	VCU	0.59	0.54	0.55	106

## Abbreviations

The following abbreviations are used in this manuscript:

AAPM	American Association of Physicists in Medicine
ASTRO	American Society for Radiation Oncology
RT	Radiotherapy
ROQS	Radiation Oncology Quality Surveillance Program
VCU	Virginia Commonwealth University
VA	Veterans Affairs
VHA	Veterans Health Administration
QA	Quality Assurance
NLP	Natural Language Processing
ML	Machine Learning
SME	Subject Matter Expert
ULMFiT	Universal Langauge Model Fine Tuning
NLTK	Natural Laguge Processing Tool Kit
RF	Random Forests
SVM	Support Vector Machines
LR	Logistic Regression
*k*NN	*k*-Nearest Neighbors
RBF	Radial Basis Function
LSTM	Long Short-Term Memory
